# Helping chromosomes and chromatids stay on track

**DOI:** 10.7554/eLife.00386

**Published:** 2012-12-18

**Authors:** Soni Lacefield

**Affiliations:** 1 **Soni Lacefield** is in the Department of Biology, Indiana University, Bloomington, United Statessonil@indiana.edu

**Keywords:** meiosis, cyclin-dependent kinase, tension, cohesin, chromosome segregation, kinetochore, S. cerevisiae

## Abstract

The prevention of premature interactions between microtubules and kinetochores is essential to ensuring that meiosis produces gametes with the correct number of chromosomes.

**Related research article** Miller M, Ünal E, Brar G, Amon A. 2012. Meiosis I chromosome segregation is established through regulation of microtubule–kinetochore interactions. *eLife* **1**:e00117. doi: 10.7554/elife.00117

**Image** Premature production of cyclins disrupts meiosis in cells

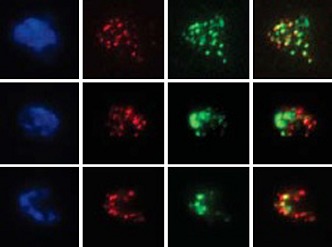

Meiosis is a process that starts with a given number of chromosomes in the nucleus of a cell, and ends with gamete cells that each contain half the number of chromosomes that were in the original cell. Most human cells, for example, have 46 chromosomes, whereas sperm and egg cells have only 23 chromosomes. In the early stages of meiosis, the process of replication copies the DNA to produce chromosomes with two sister chromatids (see Figure 1). The next stage is for chromosomes with similar sequences, called homologs, to form pairs and exchange DNA in a process called recombination. The chromosomes then undergo two rounds of segregation to complete the process. Ensuring that all these steps occur in the correct order is clearly vital for successful meiosis. Now, in eLife, Matthew Miller, Elçin Ünal and Angelika Amon of MIT, working with Gloria Brar of UCSF, reveal the mechanisms used by cells to ensure that meiosis proceeds as nature intended (Miller et al., 2012).

**Figure 1.**
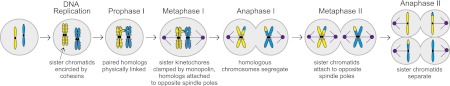
The different stages of meiosis. In this illustration the cell has two chromosomes (shown here in yellow and blue in the leftmost cell) before meiosis starts. These chromosomes are replicated to produce sister chromatids that are held together by cohesins (grey circles around the sister chromatids). During the next stage of meiosis, called Prophase I, chromosomes with similar sequences form pairs and undergo recombination, creating physical links that hold the homologs together. Next, during metaphase I, the sister kinetochores (black circles) are clamped together by a protein complex called monopolin, and the spindle microtubules (purple) attach homologous chromosomes to spindle poles (also purple) at opposite ends of the cell. Homologous chromosomes then segregate during anaphase I. During metaphase II, sister chromatids attach to opposite spindle poles and separate in anaphase II, creating meiotic products with half the set of chromosomes.

During the first round of segregation, called meiosis I, spindle microtubules attach themselves to the chromosomes with the help of large protein complexes called kinetochores that are found on each chromatid (see Figure 1). In addition to attaching the microtubules to the chromosomes, the kinetochores also correct improper attachments and move the chromosomes along microtubules. In meiosis I, the paired chromosomes segregate to opposite ends (or poles) of the spindle. In meiosis II essentially the same cast of players (that is, spindle microtubules and kinetochores), segregate the sister chromatids to produce a total of four cells. The MIT-UCSF team used budding yeast as a model to study these processes and interactions in greater detail.

Three mechanisms help ensure the proper attachment of chromosomes to spindle microtubules in meiosis I. First, during prophase, which is the first stage of meiosis I, pairs of homologous chromosomes undergo recombination. This process creates physical links that hold the homologs together, and ensures their attachment to opposite spindle poles (Brar and Amon, 2008). Second, the sister kinetochores offer only one site for microtubules to bind to: in budding yeast, for example, a protein complex called monopolin clamps the sister kinetochores together just before microtubule attachment begins. Third, protein rings made up of cohesins are thought to encircle the two sister chromatids: this creates cohesion between the chromatids and prevents them from separating prematurely during meiosis I. When the two homologous chromosomes are attached to opposite spindle poles during metaphase I (a stage after prophase), the spindle forces are resisted by the physical linkages and the cohesion between sister chromatids. Together these three mechanisms ensure that homologous chromosomes are segregated in meiosis I, while sister chromatids remain together.

The work of Miller and Ünal, who are joint first authors on the paper, and their co-workers reveals another level of regulation of meiosis I that involves proteins called M phase cyclins. When the enzyme cyclin-dependent kinase (Cdk) is bound to a cyclin, it drives cell cycle events by phosphorylating substrates (Enserink and Kolodner, 2010). Two of the cyclins that have a role in driving the cells through meiosis, Clb1 and Clb3, are transcribed at the end of prophase (Dahmann and Futcher, 1995; Chu et al., 1998; Carlile and Amon, 2008). However, if either of these cyclins is expressed prematurely, the spindle microtubules are assembled too early and, as a result, sister chromatids are segregated rather than chromosomes in a significant fraction (∼40%) of the cells (see Figure 2). This is surprising because Cdk-Clb1 is normally present (and active) during meiosis I.

**Figure 2.**
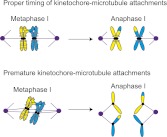
Proper timing of the interactions between spindle microtubules and kinetochores is essential for meiosis to proceed correctly. During metaphase I in normal meiosis (top), homologous chromosomes become attached to opposite spindle poles by spindle microtubules, and are then segregated in anaphase I (as shown in Figure 1). However, if the microtubules attach to the kinetochores prematurely (bottom), sister chromatids will be segregated in meiosis I, which can ultimately lead to miscarriage or birth defects in babies.

Previously Amon and co-workers have shown that the presence of the monopolin complex during mitosis (as opposed to meiosis) can clamp sister kinetochores together and lead to a meiosis I chromosome segregation pattern (Monje-Casas et al., 2007). Miller et al. now propose that for the monopolin complex to clamp sister kinetochores together, it must associate with them before they attach to microtubules. In the cells in which Clb1 or Clb3 are prematurely expressed, microtubules attach to both sister kinetochores before monopolin is active, and this leads to the segregation of sister chromatids. However, if attachment begins after monopolin becomes active, it is the chromosomes that are segregated.

To test this model, the MIT-UCSF team arrested cells undergoing mitosis after the microtubules had attached to the sister kinetochores and then induced monopolin: the sister kinetochores remained attached to the microtubules and segregated to opposite spindle poles when the cell was released from the arrest. However, if the drug nocodazole was used to depolymerize the microtubules during the arrest period, monopolin was able to clamp sister kinetochores together and almost half (48%) of sister chromatids moved to the same spindle pole. Furthermore, if the microtubules in cells that prematurely expressed Clb3 were depolymerized, the meiosis I chromosome segregation pattern was rescued. These experiments suggest that the timing of the attachment of chromosomes to microtubules is carefully regulated in meiosis to prevent premature kinetochore–microtubule interactions. And other experiments suggest that cells prevent premature interactions of kinetochores and microtubules by dismantling the outer regions of the kinetochore. Taken together all these results suggest that Miller et al have uncovered two additional mechanisms that cells use to ensure the segregation of chromosomes in meiosis I: restricting the activity of cyclin-dependent kinase bound to M phase cyclins in prophase, and also restricting the assembly of the kinetochore in prophase.

Miller, Ünal and co-workers have demonstrated that premature kinetochore–microtubule interactions lead to a mitotic pattern of chromosome segregation in meiosis I. Since this can lead to gametes with missing or extra chromosomes, which can cause miscarriage and birth defects in babies, it is crucial that we continue to improve our understanding of meiosis (Nagaoka et al., 2012). By revealing a number of hitherto unknown mechanisms used by cells to regulate the meiotic cell cycle, this work represents an important step in this quest.
